# Strigolactones Biosynthesis and Their Role in Abiotic Stress Resilience in Plants: A Critical Review

**DOI:** 10.3389/fpls.2017.01487

**Published:** 2017-08-28

**Authors:** Wajeeha Saeed, Saadia Naseem, Zahid Ali

**Affiliations:** Department of Biosciences, COMSATS Institute of Information Technology Islamabad, Pakistan

**Keywords:** Strigolactones, abscisic acid, abiotic stress, crosstalk, phytohormones

## Abstract

Strigolactones (SLs), being a new class of plant hormones, play regulatory roles against abiotic stresses in plants. There are multiple hormonal response pathways, which are adapted by the plants to overcome these stressful environmental constraints to reduce the negative impact on overall crop plant productivity. Genetic modulation of the SLs could also be applied as a potential approach in this regard. However, endogenous plant hormones play central roles in adaptation to changing environmental conditions, by mediating growth, development, nutrient allocation, and source/sink transitions. In addition, the hormonal interactions can fine-tune the plant response and determine plant architecture in response to environmental stimuli such as nutrient deprivation and canopy shade. Considerable advancements and new insights into SLs biosynthesis, signaling and transport has been unleashed since the initial discovery. In this review we present basic overview of SL biosynthesis and perception with a detailed discussion on our present understanding of SLs and their critical role to tolerate environmental constraints. The SLs and abscisic acid interplay during the abiotic stresses is particularly highlighted.

**Main Conclusion:** More than shoot branching Strigolactones have uttermost capacity to harmonize stress resilience.

## Introduction

Strigolactones (SLs) were initially known as host-derived germination stimulants for parasitic weeds from the genera *Striga* and *Orobanche*. Later on, SLs were found to be host-detection and hyphal-branching signals for arbuscular mycorrhizal (AM) fungi (Akiyama et al., 2005; Besserer et al., 2006). In addition to their original, dual role as signaling molecules in the rhizosphere, SL were further demonstrated to be a new class of branch-inhibiting phytohormones, which regulate the overall architecture of land plants (Gomez-Roldan et al., 2008; Umehara et al., 2008). This benchmark discovery in SL research led to the exponential growth of investigations on the topic in the past 8 years, which in turn opened a Pandora box of findings on biological and molecular aspects of SL activity; as more and more research groups become interested in the biological and physiological role of SL, additional functions are likely to be identified in the future. A number of authoritative reviews have appeared in the past few years, specifically covering the functions of SL in plant development and interactions with root symbionts and parasitic weeds (Ruyter-Spira et al., 2013; Waldie et al., 2014; Al-Babili and Bouwmeester, 2015; Zhang et al., 2015). Here, after a short summary of our current understanding of their biosynthesis and perception, we wish to rather report on recent breakthroughs and novel avenues of research in the area of SL functions in plant resilience and resource allocation under abiotic stress, especially emphasizing the organ-specific cross-talk between SL and abscisic acid (ABA) under drought.

## Short Overview of SL Biosynthesis and Perception

### Biosynthesis

**FIGURE 1:**
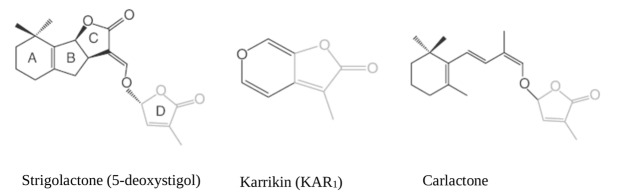
Chemical structures of Strigolactones (Xie et al., 2010; Al-Babili and Bouwmeester, 2015).

More than 20 SL and SL-like compounds have been identified so far in the root exudates of several plant species. They all share a conserved tricyclic lactone structure containing rings referred to as ABC rings, linked via an enol-ether bridge to an invariable α,β-unsaturated furanone moiety named D ring. The bioactiphore resides within the region that connects the D-ring to the core; chemical diversity is given by the stereochemistry of the B-C ring junction, the size of the A ring, and the substitution patterns of the A and B rings reviewed by (Al-Babili and Bouwmeester, 2015) see **Figure 1** for some exemplary structures. Rather wide collections of branching mutants are available in *Arabidopsis thaliana* [known as *more axillary growth* (*max*) mutants], *Oryza sativa* [*dwarf* (*d*) or *high-tillering dwarf* (*htd*) mutants], *Pisum sativum* [*ramosus* (*rms*) mutants] and *Petunia hybrida* [*decreased apical dominance* (*dad*) mutants]: their analysis was instrumental to understand and define most of the current assembly of SL biosynthetic (and signaling) pathways. Based on the observation that plants treated with inhibitors of carotenoid biosynthesis exude fewer SL from their roots, a hypothetical SL-biosynthetic pathway was initially proposed, with β-carotene as a substrate for carotenoid-cleavage dioxygenase (CCD) enzymes (Matusova et al., 2005). CCDs specifically cleave double bonds in carotenoid molecules to form carbonyl compounds called apocarotenoids (Auldridge et al., 2006). Later on, it was proven that indeed two related CCDs, CCD7 and CCD8, act sequentially in the pathway (being known, respectively, as *D17/HTD1* and *D10* in rice; *RMS5*, *RMS1* in pea, *DAD3*, *DAD1* in petunia, *MAX3*, *MAX4* in Arabidopsis) (Morris et al., 2001; Sorefan et al., 2003; Booker et al., 2004, 2005; Snowden et al., 2005; Arite et al., 2007). Rice *DWARF27* (D27) and its ortholog in Arabidopsis *ATD27*, an iron-binding, plastid-localized β-carotene isomerase, works upstream of CCD7 and CCD8 catalyzing the conversion of all-*trans*-β-carotene into 9-*cis*-β-carotene (C-40). The latter acts as substrate for CCD7 to cleave *cis*-configured carotenoids into 9-*cis*-β-*apo*-10′-carotenal (C-27) and β-ionone (C-13) (Schwartz et al., 2004; Alder et al., 2012; Waters et al., 2012a). CCD8 then acts on the C27 product of enzymatic cleavage to form a SL-like compound named Carlactone (CL), which is an intermediated compound in the SL pathway containing only A and D rings with enol ether bridge (Alder et al., 2012). CL act as endogenous precursor for more specific SLs shown in **Figure 1**, and exhibit SL like properties by inhibition of shoot branching in SL biosynthetic mutants (Rice, Arabidopsis) and promotion of seed germination *Striga hermonthica* (Scaffidi et al., 2013; Abe et al., 2014; Seto et al., 2014). The genes acting downstream to *CCD8* include *MAX1* in Arabidopsis, which encodes a cytochrome P450 of the CYP711A1clade and is responsible for the conversion of CL into functional SLs such as 5-deoxystrigol (Stirnberg et al., 2002; Booker et al., 2004; Alder et al., 2012). *MAX1* putatively converts CL into functional SL by rearrangements and modifications (hydroxylation, oxidation), converting CL to carlactonic acid (CLA) then further transformed to methyl carlactonoate (MeCLA) by an unknown enzyme (Abe et al., 2014). The latter, at least in Arabidopsis, is perceived by the SL-binding moiety of the SL receptor D14 (see Perception and Signal Transduction) and has SL-like activity, even though its carbon backbone does not fully meet the general SL structure requirements (**Figure 1**).

In rice, one MAX1 paralogue converts carlactone into *ent*-2′-*epi*-5-deoxystrigol, the presumed precursor of rice SL. A protein encoded by a second *MAX1* homolog then catalyzes the conversion of *ent*-2′-*epi*-5-deoxystrigol to orobanchol (Zhang et al., 2014), thus explaining some of the basic chemical diversity of SL (the stereochemistry at the BC-ring junction), at least in rice. Further investigation of *MAX1* and of its orthologs in Arabidopsis and rice revealed that this gene is expressed in all vascular tissues, functions only in late steps of SL synthesis, and is responsible for at least some of the structural diversity of SL (Booker et al., 2005; Umehara et al., 2010; Xie, 2016). *In vitro* association of recombinant *MAX1* with CL in yeast microsomal system confirms that *MAX1* acts as CL oxidase to convert CL stereo specifically into 9-desmethyl-9 carboxy-C2 or carlactonoic acid (CLA). Presence of both CLA and MeCLA (methyl ester carlactonoate) endogenously in Arabidopsis roots by LC-MS/MS also confirms that CL alone is the target of *MAX1.* Interestingly exogenous application of both CLA and MeCLA was found to rescue max1 mutant phenotype in Arabidopsis, however, only MeCLA act as substrate to bind with putative SL receptor of *Arabidopsis thaliana* DWARF14 (AtD14) (Abe et al., 2014). Elucidative observation were made on stereo selectivity of CL endogenously during interaction with *MAX1* by labeling experiments in rice where chemically synthesized ^13^C labeled CL was catalyzed to ^13^C- 2′-epi-5DS and ^13^C-orobanchol (Abe et al., 2014; Zhang et al., 2014). More recently, another protein was reported to act downstream of *MAX1* in Arabidopsis. Lateral Branching Oxidoreductase (LBO) is an oxidoreductase-like enzyme of the 2-oxoglutarate and Fe(II)-dependent dioxygenase superfamily, whose expression pattern partially overlaps with that of MAX3. LBO acts on *MAX1* products, such as MeCLA or CLA and converts them into an unknown SL-like compound that is hypothesized to be more effective than SL and responsible for branching inhibition (Brewer et al., 2016). For more detailed and latest discoveries in SL biosynthesis see (Al-Babili and Bouwmeester, 2015; Koltai and Prandi, 2016).

### Perception and Signal Transduction

Like most of the plant growth regulators (auxins, gibberellins, jasmonate), SL signaling mechanisms are executed by proteosomal degradation. The SL signaling machinery comprises at present the α/β-fold hydrolase named (At)D14/DAD2/RMS3, the F-box leucine-rich protein MAX2/RMS4/D3, and the D53 a repressor protein, which holds some similarity to class I CIp ATPase enzymes and belongs to a small family of proteins [SMAX1-like (SMXL)] (Stanga et al., 2013). MAX2, leucine rich F-box proteins has been shown to be part of the SKP1-CUL1-F-box-protein (SCF)-type ubiquitin ligase complex, which ubiquitinates target proteins tagging them for proteosomal degradation (Stirnberg et al., 2007; Arite et al., 2009). Interestingly, components in the auxin, gibberellin, jasmonic acid and salicylic acid perception and signal transduction machinery share similarities with either D14, MAX2 or both. The auxin and jasmonic acid co-receptors TIR1 and COI1, for example, are F-box proteins; the gibberellin receptor GID1 and the Salicylic Acid-Binding protein SABP2 belong to the α/β-hydrolase superfamily. In all of the above pathways, phytohormones act as molecular glues, allowing assembly of the active receptor/signaling complex and leading to proteasomal degradation of negative regulators of signaling (Dharmasiri et al., 2005; Ueguchi-Tanaka et al., 2007; Hamiaux et al., 2012). The current mechanistic hypothesis of SL perception implies that D14 acts in a signaling pathway mirroring one of gibberellins and including the F-box protein MAX2 (Seto and Yamaguchi, 2014). However, while GID1 does not modify its ligand, D14 is an active hydrolase; it is also a single-turnover enzyme, which explains its seemingly very low turn-over rates. The net result is that, upon binding, the hydrolysed ligand (in its bioactiphore moiety) and the receptor are locked together and thus unavailable for further perception events (de Saint Germain et al., 2016). D14 is also unique being receptor and enzyme at the same time unlike other phytohormones. D14-mediated perception of SLs depends on a catalytic triad (Ser, His, Asp) for binding and hydrolysis of SLs (Hamiaux et al., 2012). It has been proposed that MAX2/D3 acts in the SCF complex as recognition subunit for SL-loaded D14 and downstream repressors (see below). D14 in turn interacts with SLs and modifies them, mainly via its hydrophobic ligand-binding pocket and particularly the nucleophilic residues in the triad, which attack the D ring at the carbonyl of butenolide and thus separate it from the ABC part (Scaffidi et al., 2012; Zhao et al., 2013). The D14-SL complex is thought to change its conformation, which is mainly responsible for SL signal transduction. *In vitro* interaction GR24 has been shown to thermally destabilize D14 and for that catalytic triad is necessary. D14 loaded with SL is then recruited to SCF^MAX2^, which in turn directs further degradation of target proteins (e.g., D53) via the proteasome (**Figure 3**) (Jiang et al., 2013; Zhao et al., 2015). D14 itself is a target for late proteasomal degradation, which is thought to reinforce tissue desensitization to SL (Chevalier et al., 2014). It should be noted that the identity of D53 molecular partners is so far unknown, however, some orthologs have been reported in Arabidopsis (SUPPRESSOR OF MAX-2-LIKE 6-8 SMXL6-8) involved in repression of shoot branching and other SL regulated process (Soundappan et al., 2015); D53 because of the EAR motifs it contains, D53 is supposed to interact with the transcriptional corepressors termed topless-related (TPR) proteins. The D53-TPR complex could repress the transcription of targets of SL action (Jiang et al., 2013; Bennett and Leyser, 2014; Smith and Li, 2014), but this has not yet been demonstrated. Other members of the SMXL family might be in charge to modulate different aspects of SL action, while D53 seems to be the main repressor of the shoot branching effect of SL.

Several recent reports put forward interesting hypotheses on the evolution of ligand and signaling specificity by members of the wider family of D14 and D14-like proteins. The latter is a closely related protein clade to D14 proteins, some members of which have been characterized as receptors of host-exuded SL by parasitic plants, and would then represent a case of convergent evolution on SL perception with D14 (Toh et al., 2014; Conn and Nelson, 2015; Tsuchiya et al., 2015). This latter family comprises also proteins that were likely sub-functionalized to perceive other ligands in higher plants, such as karrikins – smoke-derived, D-lactone ring-containing compounds (Flematti et al., 2004; Waters et al., 2012b); both the perception of SL (in host and parasitic plants) and of karrikins require MAX2 (Zhao et al., 2015). How MAX2-mediated signaling can discriminate between different signaling pathway to generate different responses is still unknown; F-box proteins, however, are known to be promiscuous in target recruitment (Nelson et al., 2011; Nakamura et al., 2013), so the components of the signaling complex might be combinatorially assembled with different targets than D53, depending on the ligand and receptor moiety involved (Flematti et al., 2016). More aspects about SLs biosynthesis, perception, and signaling as well as structure-function relationships in the SL molecular family have been nicely addressed and updated in recent reviews (Ruyter-Spira et al., 2013; Al-Babili and Bouwmeester, 2015; Marzec, 2016; Bythell-Douglas et al., 2017; Waters et al., 2017).

## Strigolactones in Abiotic Stresses: Hormonal Interplay and Regulation

The discovery of SLs provided new opportunities in the last decade to explore hormonal regulation of plant development and acclimatization to environmental constraints. These research endeavors also identified new instances of hormonal cross talk participating to the orchestration of overall responses in plants.

Hormonal cross talk is largely at work to allow an appropriate plant response to environmental stimuli, as well as changes in architecture and acclimatization under challenging conditions such as nutrient starvation and heat/cold/salinity/light stress, by mediating growth, development, nutrient allocation and source/sink transitions.

**FIGURE 2:**
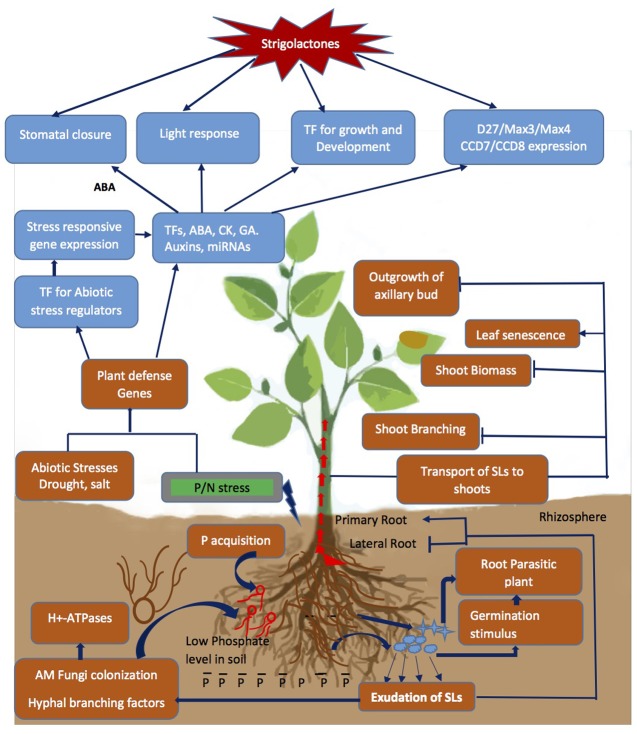
Strigolactone (SL) mediated signal transduction and changes in physiological response due to Pi depletion and abiotic stresses encountered by the plant. The P starvation alone or combined with drought/salinity leads to cascade of event from exudation of SLs to changes in above/below ground architecture of plant by cross interactions with other phytohormones and regulation of stress responsive genes, TFs and miRNAs to modulate stress tolerance. Blue arrows represent the process promoted by the SLs and capped blue lines represents repression.

Plastid-derived apocarotenoids produced in response to environmental conditions in plants include several plant growth regulators and signaling molecules besides SLs, such as retinoids and ABA. As mentioned in the Section “Introduction,” SLs have diverse biological functions in addition to regulation of branching phenotype, being important signaling compounds in the rhizosphere (**Figure 2**). Knowledge of SLs biosynthesis and their physiological role in monitoring the architecture of plants led to well established fact that SLs, need to modulate and interact with many phytohormones – particularly auxins and ABA – to exert their effect.

**FIGURE 3:**
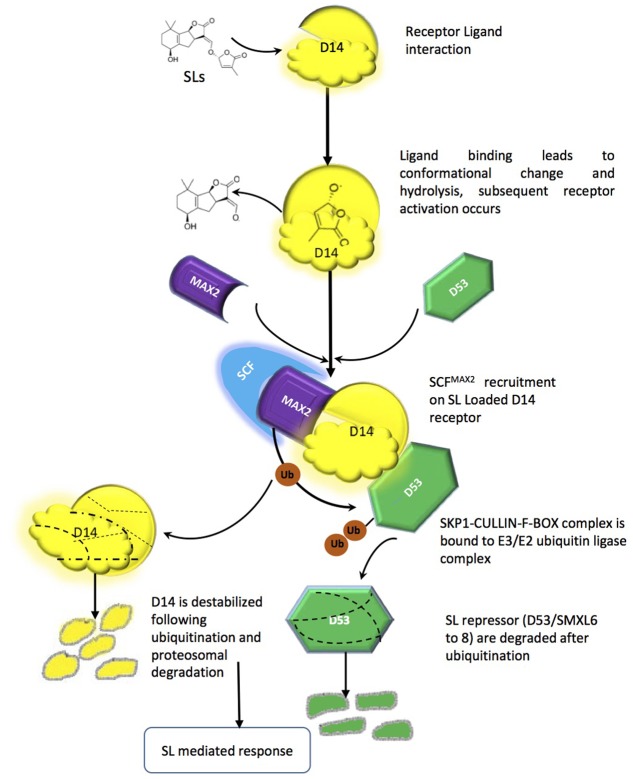
Strigolactone perception and signaling mechanism, Interaction between the ligand-binding moiety of the SL receptor complex (the α/β hydrolase D14) and the co-receptor moiety (the F-box MAX2). Such interaction promotes further binding between MAX2 and its target(s), leading to ubiquitination and degradation of the latter by the proteasome machinery and downstream signaling pathways.

Similar to other plant hormones and growth regulators, SL biosynthesis and activity is regulated by complex networks and cross talk with other hormones (Cheng et al., 2013), and recent findings suggest that SLs too, are key players of growth optimization in plants under sub-optimal environmental conditions.

The interactions among SLs and other phytohormones have been investigated in the past, especially as auxin is concerned. Auxin regulates SL biosynthesis and is involved in most of the SL-regulated developmental processes (Hayward et al., 2009; Crawford et al., 2010). For instance, SL mainly act as second messenger in shoot branching, by damping the transport of auxins in stem thereby inhibiting axillary bud outgrowth (Brewer et al., 2009). Another model for inhibition bud outgrowth proposed, SLs acts systemically to suppress PIN-FORMED (PIN) polar auxin transport protein from apical meristem thus limiting the bud growth (Bennett et al., 2006; Prusinkiewicz et al., 2009). Several lines of evidence support SL-Auxins interaction. Based on auxins and SL signaling regulatory effects on seminal root length, lateral root (LR) formation and root hair elongation (RH) became evident. Regulatory role of SLs on polarization and abundance of PIN protein in roots growth has also been indicated. In roots auxin signaling acts downstream to SLs (Brewer et al., 2009; Mayzlish-Gati et al., 2012; Sun et al., 2014). Exogenous application of synthetic auxins that are not secreted by efflux carriers (2,4-D) reversed GR24 mediated root effects in tomato while NAA and IAA failed to restore symmetric root growth and root hair (RH) elongation (Koltai et al., 2010). More recently SL biosynthetic and signaling mutants treated with exogenous GR24 showed that depletion of SL promotes lateral root formation. Both WT and biosynthetic mutants of SL in the presence of GR24 showed limited lateral root growth, however, this phenotype wasn’t evident in *max2* mutants (Kapulnik et al., 2011). In most plants SL biosynthetic genes (*MAX3* and *MAX4*) were upregulated by exogenous auxins application (Foo et al., 2005; Johnson et al., 2006; Arite et al., 2007). Role of MAX2 in auxin interaction has also been proposed under Pi deficiency, which leads to PIN2 depletion in MAX2 dependent manner. These changes in auxin efflux in SL/MAX2 dependent manner are of prime importance in changing the root architecture like increased density and length of root hair and promotion of lateral root growth due to nutrient depletion. These data suggest that SL manipulate and regulate auxins pathway either by dampening its transport or regulate perception by promoting transcription of auxins receptors TIRI reviewed by (Koltai, 2015).

Interaction of SLs with other phytohormones such as salicylic acid (SA), cytokinins (CK), gibberellins (GA), ABA have also been explored in some detail (Wallner et al., 2016). Particularly role of SL in CK and auxins in apical dominance as well as root development is emerging (Xu et al., 2015), The mechanisms of cross talk between SLs and other phytohormones (**Figure 5**) could be in the alteration in biosynthesis, sensitivity, and/or transport of either hormone (Cheng et al., 2013), whose study offers chances of better understanding hormonal regulation of plant physiology under stress. Advancements in SL based research collectively implies the hormonal interaction responsible for fine tuning the plant response, thereby emphasizing the regulatory role of SL, CK, auxins and ABA during sub-optimal environmental conditions. In the following sections, we will focus on the regulation of SL biosynthesis under abiotic stress, and on SL crosstalk with other phytohormones (particularly ABA) during abiotic stress.

### SL Production under Nutrient Starvation

Phosphate (P) and/or Nitrogen (N) deficiency in the soil is a serious abiotic source of stress and commonly encountered by most land plants. The only accessible form of P for plants in soil is inorganic (Pi) (Péret et al., 2011). Since the prime site for Pi acquisition is the roots, critical changes in plant architecture occur below ground during Pi deficiency among them are increased root growth and reduced shoot/root ratio, inhibition of shoot branching, limited primary root elongation along with extensive growth of lateral roots and root hairs (Linkohr et al., 2002; Niu et al., 2013). Consistent with the role of SLs in recruitment of AM fungi, their release and production is promoted by low soil P level (Jamil et al., 2014). Yoneyama et al. (2007a) studied orobanchol exudation form red clover (*Trifolium pratense* L.) in response to deficiency of various nutrients (P, N, K, Ca, and Mg); later on, they also tested the effects of N and P deprivation on deoxystrigol levels in sorghum (*Sorghum bicolor* [L]. Moench) exudates (Yoneyama et al., 2007b). SL quantification by LC-MS/MS showed a 20-fold increase of orobanchol in red clover exudates obtained under P starvation, while in sorghum, a 30-fold higher level of the major SL 5-deoxystrigol was reported under low P and also N. After this seminal work, production and release of SLs under P and/or N deficiency were tested in different leguminous/non-leguminous species. Most of the plants exuded more SLs in response to both P and N deficiency except tomato (*Solanum lycopersicum* L) and alfalfa (*Medicago sativa*), which only responded to P starvation (Yoneyama et al., 2007b). As more legumes and non-legumes were tested, it became clear that the ability to associate with root-nodulating, N-fixing bacteria does not correlate strictly with the response to N deficiency in terms of SL exudation. Indeed, most legumes (red clover, alfalfa, and crimson clover [*T. incarnatum* L.]) show increased SL exudation under P deficiency only, while most non-legumes respond to deficiency of both N and P; however, exceptions exist in both groups, as the legume Chinese milk vetch (*Astragalus sinicus* L.) responds to both (Yoneyama et al., 2012) while the non-legume tomato reacts only to P deficiency (López-Ráez et al., 2008a). Therefore, the ecological implications of increased vs. stable SL exudation under N deficiency in different species became unclear, even though it is now proven that SL are needed for a full nodulating response (Soto et al., 2010; Foo et al., 2013; Liu et al., 2013; De Cuyper et al., 2015), and are also able to induce surface motility (swarming) in N-fixing bacteria. It was then suggested that decreasing shoot P levels are the real triggers for SL induction under nutrient starvation; as N starvation affects shoot P levels in some but not all species equally, the effects of N deficiency on SL induction will vary depending widely on the type of plant, type of nutrient, degree of nutrient stress and macronutrient uptake strategies (Yoneyama et al., 2012, 2013). Now the fact is well documented that SLs mediated stress adaptive response are generated as a result of nutrient deficiency (Umehara et al., 2010; Jamil et al., 2011). Furthermore, while plants produce blends of SL molecules, only one or two specific types are induced under specific nutrient starvation conditions (Yoneyama et al., 2008). The ecological meaning of this selectivity is currently unknown, as remains unexplored the possibility that different SL blends have different signaling “meaning” for the producing organism and/or for the soil biota.

### SL Signaling Is Responsible for Some Plant Responses to P/N Starvation Stress

While the increased release of SLs in response to limiting P and/or N in soil has been described in many species, questions regarding the mechanisms of stress signaling leading to higher expression of SL biosynthetic proteins are still open. Umehara et al. (2015) found that rice genes involved in biosynthesis and perception (*D10*, *D17*, *D27*) of SLs were differentially regulated by P starvation/supplementation; additionally, the transcript encoding PhPRD1, the ABCG transporter responsible for exudation in soil and translocation of SLs from roots to shoot in petunia (*Petunia hybrida*) also accumulates during P starvation (Yoneyama et al., 2007a; Péret et al., 2011; Kretzschmar et al., 2012). This allows to assume that in response to low P level, the transport of SLs from the roots to the shoot increases and thus, not only root but also shoot architecture may be affected. Indeed, an increase in roots exudated orobanchol as well as the inhibition of lateral bud outgrowth was reported in WT Arabidopsis under P starvation (Umehara et al., 2010; Mayzlish-Gati et al., 2012; Yoneyama et al., 2012), which is in accordance with previous findings in rice (Jamil et al., 2011). Being Arabidopsis a non-host to AM fungi, it is hypothesized that – as it is not to the purpose of luring AM toward the P-depleted root - such increase in SLs may help to modify the architecture of plant (roots) for better utilization of available Pi see for example (Ito et al., 2015). Transcriptome analysis in alfalfa under nutrient stress also showed upregulation of 189 genes associated with AM fungal colonization/P/N deficiency, and those included SL-biosynthetic genes (Bonneau et al., 2013). Taken together, all available data point to the activation of SL metabolism under low Pi, leading to changes in nutrient allocation by inhibition of shoot branching as a survival and acclimatization strategy. At the same time, root morphology also changes under P starvation, by an increase in lateral RL and repression of PR growth. Moreover, root hair density and length increase, allowing for a larger soil-root interface and thus a more efficient Pi uptake (**Figure 2**). In general, SLs affect those features of root architecture (seminal RL, lateral root formation, RH elongation) controlled by auxins during P starvation (Pérez-Torres et al., 2008; Péret et al., 2014). SL-mediated control of root architecture during Pi depletion requires MAX2 (Mayzlish-Gati et al., 2012). Consequently, under low Pi and N conditions, SL signaling initiates morphological changes in Arabidopsis, leading to altered expression of SL biosynthetic genes (*MAX3*, *MAX4*), RH elongation, activation of P transporter genes, regulation of phosphate starvation marker genes (PSI), anthocyanin accumulation and reduction in fresh weight of plant (Sun et al., 2014; Ito et al., 2016).

### SLs and ABA Interplay

Although ABA is the most studied stress-responsive hormone, the individual role of ethylene, CKs, BRs, and auxins during environmental stress is emerging, as is the impact of their mutual cross-talk (Fujita et al., 2011; Arc et al., 2013; Fahad et al., 2015; Wani et al., 2016). SLs as well, were recently shown to play a prominent role in abiotic stress responses (see SL Signaling is Responsible for Some Plant Responses to P/N Starvation Stress and below), and we have thus entered a new phase in which their interaction with other phytohormones in the frame of abiotic stress resistance is being targeted experimentally. ABA is sometimes referred to as the stress responsive growth regulator *par excellence*, due to its role in stomatal closure and, in some plants, as long-range signal triggered by abiotic stresses like drought, desiccation, salinity, pathogen attack and wounding (Zeevaart and Creelman, 1988; Davies et al., 2002). Changes in ABA levels induce expression of many genes either directly or indirectly by upregulation/downregulation of transcription factors (Chandler and Robertsonz, 2003). During dehydration indeed, ABA accumulation in guard cells leads ultimately to stomatal closure. *De novo* synthesis of ABA in stressed leaves and roots is also reported (Boursiac et al., 2013). Changes in level of ABA also takes place via influx through transporters, Reactive oxygen species (inactivate conjugates). Peculiar increase in ABA accumulation in guard cell are mainly accounted for dehydration stress related rapid response, while long term soil water deficit and drought conditions are circumvented by ABA synthesis in main vasculature (vascular parenchyma, plastid, cytosol) (Merilo et al., 2015). These sudden changes in ABA levels plus its ability to transport over long distance peculiarly act as stress messenger. This also involve changes in long distance transport of ABA as well as modulation of ABA in guard cell leading to cascade of reactions for stomatal closure, reduce leaf expansion, promotion of root growth and prevent loss of water (Hong et al., 2013). Apart from stomatal closure as a mean to cope with stress, changes in ABA level also trigger transcriptional activation of genes encoding protein required for stress tolerance in plants, these include osmoprotectants, dehydrins, salinity and drought related genes (Wilkinson and Davies, 2002; Fujita et al., 2011).

**FIGURE 4:**
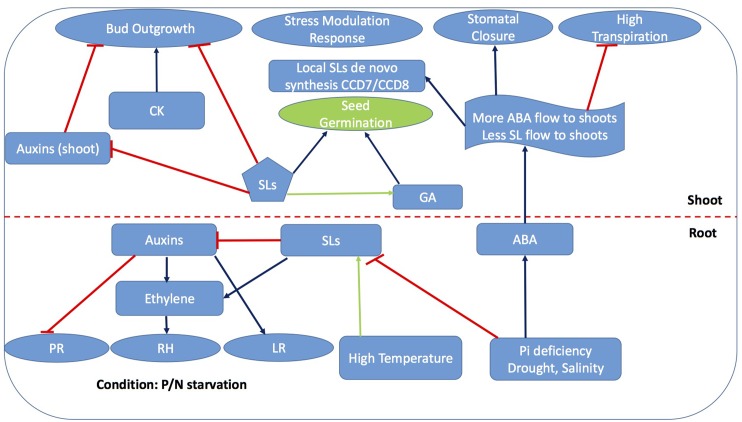
Strigolactone-ABA cross linking platforms most of the phytohormones interactions for stress mediated response. Blue arrows represent activation of process while capped red lines represent repression while green arrows represent partially explored process promoted by SLs. PR, Primary roots; RH, Root hair; LR, Lateral roots; GA, Gibberellic acid; CK, *Cytokinins*

**FIGURE 5:**
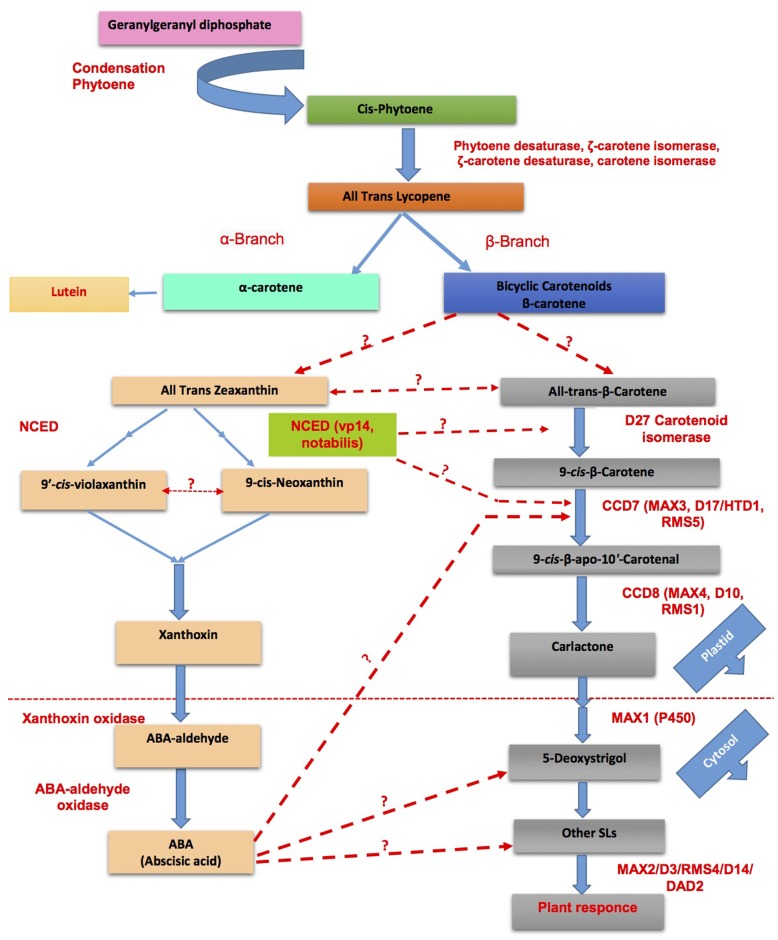
Cross talk between SLs and abscisic acid (ABA) biosynthesis for the adaptability of plants in response to challenging environmental conditions (modified from Ruyter-Spira et al., 2013; Zhang et al., 2013). Dotted lines represent the unexplored/partially explored interactions.

Strigolactones have emerged to be key players in plant physiology under stress such as upon nutrient starvation (see SL Signaling is Responsible for Some Plant Responses to P/N Starvation Stress) but also drought, salinity (Liu et al., 2013; Bu et al., 2014; Ha et al., 2014; Visentin et al., 2016), and light stress (Gonzalez-Perez et al., 2011; Jia et al., 2014). ABA accumulates in plants due to the activity of enzymes in the 9-*cis*-epoxycarotenoid dioxygenase family (NCED), catalyzing the cleavage of 9-*cis*-epoxycarotenoids to xanthoxin, i.e., the first step in the ABA biosynthetic pathway (Cutler and Krochko, 1999; Bouwmeester et al., 2007). Since the beginning of SL research, the shared carotenoid precursor of SLs and ABA have intrigued the possible crosstalk of SLs at various level of biosynthesis which have been partly explored (**Figures 4**, **5**). Although the initial working hypothesis was that both hormones interact with each other at the biosynthetic level, and that induction of ABA biosynthesis impacts SLs formation and vice versa by substrate competition, recent work has highlighted more subtle interactions.

Root extracts and exudates of ABA-deficient maize plants having null mutations in the ABA-biosynthetic gene *ZmNCED1* induced significantly less germination of the parasitic seeds, an effect that may be associated with low levels of SLs (Matusova et al., 2005; López-Ráez et al., 2008b). In a more recent report, ABA was shown to induce *MAX3* and *MAX4* transcript accumulation in Arabidopsis (Ha et al., 2014). These results collectively highlight a regulatory role for ABA in SL production, disfavouring the hypothesis of a direct involvement of NCEDs in SL production and of course, of the carotenoid substrate being limiting for the two competing pathways. Since SLs are also involved in AM fungal colonization and involvement of ABA in AM colonization has also been extensively investigated, the SL-ABA crosstalk may have implications in this area of research, too. Aroca et al. (2008) reported that lettuce plants colonized by AM fungi can modulate their endogenous ABA so to better cope with drought stress than non-mycorrhized plants. In tomato, ABA-deficient *sitiens* mutants having only 8% ABA as compared to wild-type, were less susceptible than the latter to AM fungal infection (Herde et al., 1999). Endogenous ABA is reported to enhance the spread of fungi as well as arbuscule development in tomato, while exogenous ABA treatment increases the rate of colonization (Herrera-Medina et al., 2007). These reports therefore suggest a role for ABA in controlling colonization by AM fungi, which may depend on underlying interactions with other plant hormones. For example, it is well established that ABA/GA interaction with regulate various growth and developmental processes including AM colonization in tomato (Martín-Rodríguez et al., 2016). Since SL signaling mechanism has revealed surprising overlapping mechanism with GA pathway (Wallner et al., 2016). In such an interlinked context, the effects of ABA and SLs on AM fungi infection cannot be easily made apart (López-Ráez, 2016). The reduction of SL levels in the above-mentioned tomato mutants *sitiens* deficient in ABA could be the reason of low AM colonization; however, CCD8-silenced transgenic tomato was not significantly affected in AM colonization, indicating that the residual SL production in such lines might be enough to warrant efficient host perception by AM fungi (Kohlen et al., 2012). Also, there are hints – e.g., in lettuce – that not only AM colonization is affected by ABA and SLs, but that levels of the latter can be altered by AM colonization, also in dependence of water availability in soil (Aroca et al., 2013; Ruiz-Lozano et al., 2016).

A separate analysis should be dedicated to the converse effect – i.e., of SLs on ABA levels – and to the SL-ABA cross-talk under abiotic stresses. In the first report of SL-ABA interaction by López-Ráez et al. (2010), blocking ABA synthesis by specific NCEDs inhibitors reduced the synthesis of 3 major SLs in tomato when compared to untreated wild type, along with a slight reduction of ABA in roots. Interestingly, tomato plants treated with an inhibitor of CCDs (among which, the SL biosynthetic enzymes CCD7 and CCD8 had unchanged ABA concentrations in roots, suggesting that while ABA positively regulates SL content, the opposite may not be true (López-Ráez et al., 2010). However, under drought or combined osmotic/low P stress, SL-deficient tomato or Lotus plants show significantly reduced ABA levels in their shoots (Liu et al., 2015; Visentin et al., 2016). In a following report by Ha et al. (2014), Arabidopsis SL biosynthetic and signaling *max* mutants were subjected to drought and salinity stress to investigate the involvement of SLs in abiotic stress resilience. No significant differences in ABA levels could be detected between wild-type and SL mutants in this species, but stressed tissues were not analyzed; this and the previous datasets on tomato and Lotus collectively suggest a general positive influence of SLs on ABA levels under stress, possibly with some species-specific variability. Nonetheless, both SL-deficient and perception mutants of Arabidopsis were reported as more stress-sensitive at different developmental stages in the same work. The positive role of SL in abiotic stress resistance was further proven by exogenous application of SL at the shoot level, which improved performances under stress both of wild-type and of SL-deficient mutants, but not of SL signaling mutants. Interestingly, all SL mutants were partially insensitive to exogenous ABA as compared to wild-type plants, both at germination and seedling developmental stage (Ha et al., 2014). Sensitivity of the signaling mutant *max2* in Arabidopsis to abiotic stresses as reported by Ha et al. (2014) was somewhat confirmed by Bu et al. (2014), whose conclusion on hypersensitivity of SL signaling mutants to dehydration and elevated transpiration rate due to reduced stomatal closure as compared to wild-type are in agreement with the above results. However, besides this shared conclusion, the two reports contradict each other at various levels. On one hand, Ha et al. (2014) reported that exogenous ABA negatively affects seed germination and development of wild-type seedlings but not of the SL mutants *max3, max4* and *max2*. On the other hand, *max2* displayed hypersensitivity to ABA at pre- and post-germination stages in the work by Bu et al. (2014). More strikingly, SL-depleted Arabidopsis plants were reported as hypersensitive to stress in Ha et al. (2014), while Bu et al. (2014) found a similar phenotype only in the signaling mutant *max2*, thus excluding a direct role for the SL metabolites in osmotic stress responses. However, the contribution of SLs in osmotic stress resistance was confirmed in other species, thus supporting the results by Ha et al. (2014) in Arabidopsis. Liu et al. (2015) reported that SL-depleted *Lotus japonicus* was more stress-sensitive than its wild-type counterpart; more recently, the same was proven in tomato (Visentin et al., 2016). In all three species, the effect was linked to partial insensitivity to endogenous and exogenous ABA. These datasets certainly show that endogenous SLs give an important positive contribution to stress resistance by increasing ABA sensitivity. This, coupled to lower ABA contents than in the wild-type, would certainly contribute to poor performances of SL mutants under drought. Altogether then, the drought-hypersensitive, ABA-hyposensitive phenotype shown by SL-depleted plants (tomato, Lotus, Arabidopsis) is persuasive proof that during stress, proper ABA accumulation and functioning at the guard cell level requires intact SL metabolism and signaling.

If SLs contribute to drought resistance one would expect their levels to increase under stress. This may be true in shoots, where although metabolites remain under the detection threshold, the transcript of biosynthetic genes are more concentrated in dehydrated than unstressed wild-type tissues, both in Arabidopsis and tomato (Ha et al., 2014; Visentin et al., 2016). However, surprisingly, the opposite is true for the main site of SL production under normal conditions, i.e., the roots. There, both the transcript of genes involved in SL biosynthesis and exudation, and the metabolites themselves in tissues and exudates, were markedly decreased by drought or salinity in non-mycorrhized tomato (Ruiz-Lozano et al., 2016; Visentin et al., 2016) and by osmotic stress in Lotus (Liu et al., 2015). In the latter set of experiments, even the increase of SL exudation triggered by P starvation alone was reversed to a sharp decrease under combined osmotic/low-P stress, indicating that in the case of multiple stresses, response to one can override the other.

The above-mentioned organ-specific dynamics of SL production during dehydration were investigated into more detail in tomato. The hypothesis of a possible role for the drought-triggered SL decrease at the root level in long-distance signaling of stress to the shoots was tested, by comparing the eco-physiological performances and ABA content of wild-type and SL-depleted plants with those of plants obtained by grafting wild-type shoots on SL-depleted roots, in a drought time-course (Visentin et al., 2016). This latter genotype combination was meant to mimic, as far as SLs are concerned, the hormonal balance typical of stress even in unstressed conditions – i.e., low SLs in the roots but normal SL metabolism in the shoot. The results show that under some respects (low gas exchange rates and high transcription of SL-biosynthetic genes in the leaves), such hetero-grafted plants behave as if they were indeed under mild stress, and thus, that low SL levels in the roots can, alone, signal distress at the root level. ABA content is, however, either unchanged or decreased in wild-type shoots grafted onto SL-depleted roots as compared to wild-type shoots joined to wild-type roots. The phenotype of hetero-grafted plants is likely explained, mechanistically, by their shoot hypersensitivity to endogenous and exogenous ABA, in terms of stomatal closure. This, in turn, might be due to the increased SL levels in shoots as suggested by the up-regulation of SL-biosynthetic genes; treatment with exogenous SLs is indeed sufficient to increase sensitivity to ABA (Visentin et al., 2016). In the roots, the SL-ABA interactions might be different than in shoots: keeping SL levels high by a pre-treatment with exogenous SLs hampers the osmotic stress-triggered ABA increase in Lotus roots. Notwithstanding that such peculiar SL-ABA relationship should be verified in other models, this means that SLs repress ABA synthesis in the roots and thus, that their levels must drop also to allow local ABA build-up upon stress. This in turn may be needed both for local and systemic stress signaling, assuming shootward transport of ABA (Liu et al., 2015). It must be noted here that while it is known that shootward translocation of root-synthesized ABA is important for systemic signaling of stress in some plant species (Manzi et al., 2015), it is not in all plants. Tomato shoots, for example, do not need root-produced ABA in order to react appropriately to water scarcity in soil (Chen et al., 2002).

## Conclusion and Future Prospective

The SL-ABA cross-talk has been increasingly spotlighted by research aimed at dissecting and understanding abiotic stress tolerance; interest is escalating because of the theoretical possibility of engineering phytohormonal signaling and metabolism for the improvement of crop performances under natural stress conditions. If we really are to do so, and exploit SL biology to the purpose of sustainable agriculture, the most immediate challenge ahead is to understand the molecular underpinnings of the pervasive SL effects on plant phenotypes under stress, as well as how they may connect physiological strain to appropriate development progression. How do SLs modulate ABA sensitivity? Is their effect linked to altered efficiency or abundance of components in the ABA signaling machinery, and/or to a modulation of ABA transport? Data in Arabidopsis, Lotus and tomato indicate that in the former, ABA transporters are down-regulated in SL-depleted vs. wild-type leaves under drought (Ha et al., 2014); while in the latter two, SL-defective leaves retain the ability to close their stomata comparably to the wild-type in response to exogenous ABA fed through the petiole, if given sufficient time (Liu et al., 2015; Visentin et al., 2016). Both observations support some contribution to the final phenotype of SL-depleted plants, for less efficient ABA translocation. Also, is the effect on sensitivity mutual, i.e., is ABA able to affect sensitivity to SLs, and/or their translocation? Is there a connection between SLs and regulatory molecules such as miRNAs (Marzec and Muszynska, 2015), as proven for other hormones (Curaba et al., 2014; Ferdous et al., 2015) and if so, is this connection relevant for SL effects under drought? Finally, based on the newly acquired knowledge, can we envisage breeding or management strategies that can move agriculture one step closer to sustainability, for example by exploiting the drought-tolerant phenotype of heterografted tomato plants? As reported by Visentin et al. (2016).

## Author Contributions

WS has compiled the literature and written the manuscript. ZA has initiated and supervised the research. ZA and SN have helped in compilation of manuscript. All three authors have edited and revised the approved manuscript submitted. WS and SN contributed equally in this manuscript.

## Conflict of Interest Statement

The authors declare that the research was conducted in the absence of any commercial or financial relationships that could be construed as a potential conflict of interest.
